# Nutrition Messaging by Healthcare Students: A Mixed-Methods Study Exploring Social Media Usage and Digital Competence

**DOI:** 10.3390/nu16101440

**Published:** 2024-05-10

**Authors:** Hüsna Kaya Kaçar, Ömer Furkan Kaçar, Fiona McCullough

**Affiliations:** 1 Division of Nutrition and Dietetics, Faculty of Health Sciences, Amasya University, Amasya 05100, Türkiye; husna.kacar@amasya.edu.tr; 2 Doctoral School of Health Sciences, Faculty or Health Sciences, University of Pécs, 7622 Pécs, Hungary; 3 Department of Biochemistry and Medical Chemistry, Medical School, University of Pécs, 7624 Pécs, Hungary; 4 Division of Food, Nutrition and Dietetics, School of Biosciences, University of Nottingham, Nottingham NG7 2TU, UK; fiona.mccullough@nottingham.ac.uk

**Keywords:** digital competences, education, healthcare students, nutrition communication, social media

## Abstract

Aim: The COVID-19 pandemic leads to a significant digital transformation in higher education and healthcare practices. This study aimed to investigate the level of digital competence, views and experiences, social media usage, and perceived barriers to digital communication among healthcare students. Method: Employing a mixed-methods approach, quantitative data were gathered through an online survey, while qualitative insights were gleaned from semi-structured questionnaire responses obtained during focus group discussions. A total of 143 nursing and midwifery students from Turkey, along with 54 dietetics students from various European countries, participated in the study. Results: A significant proportion of nursing (43.5%) and midwifery (55.2%) students advocated for integrating digital technology training into university curricula. Instagram has emerged as the predominant platform for sharing healthcare/nutrition information among students. However, concerns have been raised regarding the prevalence of “before/after” posts on social media promoting weight loss, which were identified as low-quality content by participants. Conclusions: These findings underscore the importance of integrating digital technologies and social media into healthcare, nutrition education, and practice. Additionally, there is a pressing need to establish professional and ethical standards for digital nutritional communication. By addressing these challenges, educators can better equip healthcare students to navigate the complexities of modern healthcare practices and enhance patient-care outcomes.

## 1. Introduction

The COVID-19 pandemic has not only disrupted traditional face-to-face learning but also propelled a significant digital transformation in higher education globally, as evidenced by studies [1,2]. Simultaneously, healthcare and dietetic practices have shifted towards virtual platforms, including telemedicine/telenutrition and remote patient monitoring, indicating a broader trend towards digitalization [3,4]. This shift underscores the critical integration of healthcare education and nutrition training into the digital landscape.

Moreover, within the education sector, there’s a notable challenge to the traditional approach of gaining competitive advantage, attributed to global phenomena such as digital transformation, globalisation, and the pervasive influence of social media [5]. The pandemic has further accelerated the adoption of e-learning, prompting investigations into factors like computer anxiety and digital readiness among medical students [6]. Notably, research indicates that gender, age, and internet usage significantly impact these factors, with increased internet use correlating with enhanced digital readiness and subsequently influencing perceptions of academic performance.

Social media is a key dimension of digital transformation and can serve several purposes in both healthcare and dietetic practice by providing a platform to communicate about health, food, and nutrition [7,8]. The benefits of social media include accessibility and widening access to health information for various demographic groups compared to traditional methods of communicating; however, the quality and reliability of the information that is shared are highly variable and therefore should be monitored [7]. A study that investigates how gender and social networking sites influence higher education students’ educational social media behaviours showed gender differences in content creation while highlighting social networking site preferences for educational use, such as Instagram, WhatsApp, and YouTube [9]. These findings shed light on students’ social media behaviours and underscore the importance of understanding gender differences in social media usage within educational contexts.

In contemporary healthcare education, digital literacy and effective communication skills are crucial. However, despite widespread recognition of their importance, there remains a notable gap in understanding the specific needs and challenges faced by healthcare students, particularly those in nursing, midwifery, and dietetics. This study aimed to bridge this gap by investigating various aspects, including the level of digital competence, views, experiences, social media usage, and perceived barriers to digital communication among students in these fields. Our research adopted a mixed-methods approach, combining quantitative data collected through an online survey with qualitative insights from focus groups. This methodological choice would allow for a comprehensive understanding of the digital landscape within healthcare education, capturing both the breadth of quantitative data and the depth of qualitative insights. Quantitative data provide statistical analyses to identify trends and patterns, while qualitative insights offer nuanced perspectives and context to enrich our understanding of students’ experiences and perceptions. Embracing this mixed-method approach allows for a holistic exploration of complex phenomena, providing a more comprehensive understanding than either approach alone could achieve.

The study’s scope encompassed a diverse cohort of undergraduate and graduate students from healthcare programmes across Turkey and European countries. This diversity ensures representation from varied educational backgrounds and cultural contexts. Ultimately, the findings of this research provide implications for healthcare education, practice, and policy, particularly in light of the ongoing digital transformation of the healthcare sector.

## 2. Materials and Methods

### 2.1. Study Design

A mixed-methods approach was employed to comprehensively investigate the level of digital competence, views, experiences, social media usage, and perceived barriers to digital communication among students of nursing, midwifery, and dietetics. This methodological framework comprises both quantitative and qualitative methodologies to ensure a nuanced exploration of the subject matter.

### 2.2. Participant Recruitment

The recruitment strategy entailed a multi-faceted approach tailored to the diverse backgrounds and geographical locations of the participants. Nursing and midwifery students were specifically targeted at Amasya University, leveraging a web link disseminated via email. Meanwhile, dietetic students were sourced from various European countries through the extensive network of the European Federation and Association of Dietitians (EFAD) mailing list. Additionally, the survey was promoted via the social media channels of EFAD and ENDietS (the European Network of Dietetic Students). For the focus group component, nursing and midwifery students were provided with the option to participate at the conclusion of the survey, while dietetic students were invited via social media platforms frequented by nutrition and dietetics students in Turkey.

### 2.3. Data Collection Instruments

The core data collection instruments included an online descriptive survey and semi-structured questionnaires utilised during virtual focus groups. Developed by EFAD, the survey encompassed a blend of open-ended and closed-ended inquiries meticulously designed to capture the multifaceted dimensions of digital healthcare/nutrition communication. Embracing the Technology Acceptance Model, the survey sought to assess the propensity of students towards the utilisation and sustained engagement with digital communication platforms for disseminating healthcare/nutrition-related information.

The focus group sessions were conducted virtually over the Zoom Meeting application, with participants providing consent for video recording. These recordings were transcribed verbatim to ensure accuracy in capturing participants’ responses and discussions. In addition to audio recordings, researchers took detailed field notes to capture non-verbal cues and contextual information during the virtual discussions. These recordings and field notes served as primary sources of data for thematic coding and analysis. By employing these data collection methods and ensuring transparency in our approach, it was aimed to enhance the reliability and validity of the findings.

### 2.4. Data Analysis

Quantitative data obtained from the online survey underwent analysis employing statistical tools such as descriptive statistics and frequency measures, facilitated by SPSS software v22. On the other hand, qualitative data gathered from the semi-structured questionnaires administered during focus groups was subjected to a meticulous manual analysis. This involved employing a thematic analysis approach based on grounded theory.

### 2.5. Ethical Considerations

The research protocol secured ethical approval from the School of Sociology and Social Policy Research Ethics Committee at Nottingham University, United Kingdom, in May 2021, bearing the accreditation RN: 2021-060. 

Prior to their involvement in any aspect of the research, participants were provided with detailed information regarding the nature, purpose, and potential implications of their participation. This included elucidation on the voluntary nature of their involvement, assurance of anonymity and confidentiality, and delineation of their rights as research subjects. Participants were explicitly informed of their right to withdraw from the study at any point without repercussion. Consent was obtained through electronic means for the online survey, with participants required to affirmatively acknowledge their consent before proceeding. Similarly, verbal consent was obtained from participants prior to their participation in virtual focus groups, with researchers ensuring that participants were fully apprised of the study’s objectives and procedures. The meticulous adherence to principles of informed consent underscored the commitment to upholding the dignity, autonomy, and welfare of all participants throughout the research process.

## 3. Results

### 3.1. Participants’ Characteristics

Participants were recruited from Amasya University in Turkey, where the principal investigator was affiliated, as well as from a European dietetics student network. The study comprised 143 participants from Amasya University, with 85 enrolled in the Division of Nursing and 58 in the Division of Midwifery. Additionally, 54 dietetic students from various European countries participated in the study. Predominantly, the participants were female, constituting 78.8% from nursing, 100% from midwifery, and 83.3% from dietetics. Furthermore, the majority of participants fell within the 20–24 age bracket, comprising 67.1% of the nursing group, 87.9% of the midwifery group, and 64.8% of the dietetics group. Notably, all nursing and midwifery students were from Turkey, whereas the dietetic students hail primarily from Spain (48.8%), followed by Turkey (16.7%), and Greece (7.4%).

### 3.2. Digital Competences

In terms of university training aimed at fostering digital competency among students for their future placements, a significant proportion of nursing (43.5%) and midwifery (55.2%) students concurred that “students should receive training at university about digital technologies in healthcare practice”. However, a notable majority expressed dissatisfaction with the adequacy of training received on digital technologies in clinical practice during their university education. Key areas of digital training offered at the university included the ability to discern trustworthy health information online, the utilisation of social media for health communication purposes, and proficiency in email usage. Academic classes and lectures emerged as the primary sources of such information, as indicated by the majority of students (Table 1).

The majority of nursing and midwifery students reported a desire to enhance their ability to identify and install software, programs, and applications for better healthcare practice (57.6% of nursing students and 69.0% of midwifery students). Additionally, there was a keen interest among students to improve their competencies in evidence-based implementation of digital tools in healthcare practice, with 56.5% of nursing students and 67.2% of midwifery students expressing this idea. Conversely, only a minority of nursing (17.6%) and midwifery (15.5%) students expressed a desire to develop skills for creating infographics. These categories/topics were defined in the survey instrument (Table 2).

Dietetic students conveyed a distinct set of training preferences, with a notable proportion expressing interest in training on Google Analytics (27.8%), communication strategies and digital collaborations with other experts (25.9%), and professional standards for dietitians in the digital environment (22.2%). These insights underscore the diverse and evolving training needs of healthcare students as they navigate the digital landscape of their respective disciplines.

### 3.3. Digital Communication Channels for Communicating Healthcare/Nutrition Information

Forty-three percent of nursing students reported occasional use of digital communication channels to disseminate healthcare information, while 55.2% of midwifery students and 11.1% of dietetics students indicated utilising these channels for sharing healthcare/nutrition-related content. Primary reasons cited for refraining from online dissemination of healthcare/nutrition information included challenges such as limited internet connectivity (20.0% of nursing students, 22.4% of midwifery students), lack of technical proficiency (17.6% of nursing students, 31.0% of midwifery students), and time constraints (10.6% of nursing students, 12.1% of midwifery students, and 5.6% of dietetics students).

Among the digital communication platforms, Instagram emerged as the preferred choice. While 31% of nursing and 7% of dietetics students reported moderate posting activities, a total of 59% of midwifery students indicated low posting frequency on Instagram. Notably, nursing and midwifery students predominantly utilised personal Instagram accounts, whereas dietetics students leveraged professional accounts for disseminating healthcare/nutrition information (Table 3).

When queried about their motivations for utilising digital platforms to communicate nutrition information, dietetics students cited various drivers, including “to share evidence-based nutrition information” (16.6%), “to make nutrition information actionable for the public” (14.8%), “to improve my personnel branding/promotion” (13.0%), and “to keep myself up to date” (13.0%).

Furthermore, when prompted to suggest strategies for combatting misleading nutrition information online, dietetics students advocated for the provision of evidence-based facts (33.3%) and public education initiatives aimed at guiding individuals in discerning trustworthy nutrition information (33.3%). These findings underscore the multifaceted roles and responsibilities of dietitians in navigating the digital realm and fostering informed decision-making among the public.

### 3.4. Focus Groups

Two online focus groups were conducted using the Zoom Meeting application to facilitate rich discussions among participants. The initial focus group comprised nursing (n = 4) and midwifery (n = 4) students, consisting of a diverse mix of one male and seven female participants. The session, lasting 45 min, provided sufficient time for in-depth exploration of key topics. Subsequently, a second focus group was convened exclusively with dietetic students (n = 5), extending over 55 min. The composition included one male and four female students, with participants spanning various stages of their dietetics education, ranging from the second to the final (i.e., fourth) year.

Analysis of transcripts from both focus groups revealed three overarching themes that encapsulated the essence of the discussions: virtual consultations, social media, and strategies to improve the digital competence of healthcare workers/dietitians. These themes emerged organically from the collective insights and perspectives shared by participants, offering valuable insights into the evolving landscape of digital healthcare practices and the aspirations of future healthcare professionals.

**Theme 1: Virtual consultations**

The majority of students found virtual consultations useful for some critical processes, such as palliative care and pregnancy. The comment below illustrates a possible advantage of virtual consultation, which is not currently conducted as part of palliative care in a state hospital. A dietetics student who underwent placement in a state hospital expressed the following:

*“When the patient from palliative care refers to us (dietetic department), dietitian plans his diet based on the information on his folder without seeing the patient and gives it (diet list) to the patient’s companion. We (dietitians) explain the diet to the patient’s companion and ask him (the patient’s companion) to explain it (diet list) to the patient…… I think it would be much better if we could communicate directly (virtually) with the patients who could not access in person”*. Dietetics student, year 3.

Another student considered virtual consultations for pregnant women to be useful.

*“There were pregnant women who could not come to the clinic from time to time, we were communicating with them via WhatsApp, and it was useful”*. Dietetics student, year 4.

Nursing and midwifery students’ views on virtual consultation were different from dietetics students. The midwifery student said the following:

*“Online events are very useful during this period; we can even participate in international events without travelling. However, I do not find distance education and consultancy services effective. For example, we provide breastfeeding counselling to pregnant women, but when we do this online, it is not effective. In-person counselling is more beneficial”*. Midwifery student, year 4.

**Theme 2: Social media**

Opinions about the use of social media by healthcare workers and dietitians included the following: 

**High-quality posts**. The characteristics of high-quality social media posts were identified by the students as follows:

- Including references;
- Including general healthy eating advice;
- Including high-quality images.

*“If it (social media post) includes a reference or is based on a scientific fact, it seems reliable to me”*. Dietetics student, year 3.

*“The posts by a dietitian should not go beyond general healthy eating recommendation such as water intake, consumption of fresh fruits and vegs etc.”*. Dietetics student, year 4.

*“I think the harmony of visual and post caption is important. If a sleezy image uses, what the caption is not very important to me…. Attracting people’s attention with a good image is significant”*. Dietetics student, year 4.

**Low-quality posts.** The following characteristics of low-quality social media posts were identified by the students: 

Before/after posts:

- Shock and very-low-calorie diets and detox recipes;
- Posts with irrelevant hashtags;
- Long and too much information;
- Posts with irrelevant images;
- Personal posts over a professional account.

*“‘This shock diet makes you lose 10 kilos in 2 days!’ This is done by a dietitian! We may encounter very strange and absurd titles and content just to increase the number of followers and interaction”*. Dietetics student, year 3.

*“They (dietitians) constantly share low-calorie recipes. This seems strange to me. They are sharing low-calorie recipes by constantly adding honey or dates instead of refined sugar into things. I found many dietitians’ post useless especially on Instagram”*. Dietetics student, year 3.

*“If a dietitian uses irrelevant hashtags to become more popular and gain more followers, the post seems low quality to me”*. Dietetics student, year 4.

*“I see that some dietitians share their photos and their private lives in order to reach more followers on their professional accounts. From time to time, they give information about nutrition under their own photos. I think that the personal account and the professional account should be separated”*. Dietetics student, year 3.

**Theme 3: Strategies to improve digital competences**

Having recognised inappropriate and low-quality social media posts by dietitians, the students provided some recommendations that could improve the digital competence of healthcare workers and dietitians. The majority of students agreed with the statement that digital technology content should be included in the university curriculum.

*“In fact, it should be included as a course (module) in nutrition and dietetics undergraduate education to increase this competence (digital) and use social media more accurately”*. Dietetics student, year 4.

This view was echoed by another student.

*“The subject of rhetoric, ethical rules and digital competences such as video editing should be included in this module”*. Dietetics student, year 3.

A nursing student’s comment was about the electronic patient registration system, and they stated the following:

*“There is an electronic registration system used in hospitals and we spend a lot of time learning this registration system after we are appointed. There is a computer module at university, but I am still very bad at computer because I do not think that computer education is enough. I especially think that electronic patient record system should be taught in school. I worked at the hospital for a while since I graduated from a medical career collage and I spent at least two weeks to learn this system, and I had to constantly consult someone while learning”*. Dietetic student, year 1.

## 4. Discussion

This study employed a mixed-methods approach to explore the digital competences, perspectives, and recommendations regarding digital communication and social media among nursing and midwifery students from Turkey, as well as dietetics students from various European countries. The findings revealed a consensus among nursing and midwifery students regarding the necessity of digital technology training within healthcare education curricula, with many expressing dissatisfaction with the current level of training received. Notably, Instagram emerged as the preferred digital communication channel across all student groups, with nursing, midwifery, and dietetics students alike gravitating towards its versatile platform. Interestingly, while nursing and midwifery students predominantly favoured personal Instagram accounts for communication purposes, dietetics students exhibited a preference for professional accounts when disseminating healthcare/nutrition information. Moreover, students highlighted the importance of integrating digital competence into university curricula, emphasising the need for comprehensive training to prepare them for the evolving demands of digital healthcare practice. These findings highlight the imperative for healthcare education institutions to reevaluate their approach to digital literacy training, ensuring that students are equipped with the requisite skills to navigate and harness the potential of digital technologies in their future professional endeavours.

Our study highlights the urgent need for comprehensive digital skills and technology training in healthcare education, echoing the sentiments expressed in established guidelines and recommendations. The EFAD academic standards curriculum guidance outlines basic requirements for the effective implementation of a training programme [10]. According to this guideline, students are expected to possess the ability to proficiently utilise computer software to bolster their professional practice, leverage digital tools for effective communication, and discern the advantages and risks associated with utilising social media in a healthcare context. Similarly, NHS England (2019) recommended that “education providers must ensure that students gain an appropriate level of digital literacy at the outset of their study for their prospective career pathway” to support a digitally enabled health system. Despite the resonance of these guidelines with the views expressed by the majority of students in our study, there was a prevailing sentiment among participants that adequate training in digital technologies for healthcare practice was lacking at the university level. 

A study examining the self-reported digital literacy levels of nursing students (n = 84) at an Australian university revealed a notable disparity between students’ proficiency in everyday digital settings and their ability to translate these skills into practice within a healthcare context [11]. Consistent with these findings, both quantitative and qualitative results of our study underlined the imperative of incorporating digital technology content into university curricula to enhance students’ digital competences, as stated by the students in the focus groups. These insights present the critical importance of aligning educational curricula with the evolving demands of digital healthcare practice to ensure the preparedness of future healthcare professionals.

In our study, Instagram emerged as the predominant digital communication channel among nursing, midwifery, and dietetics students, reflecting its widespread popularity among university populations. This finding resonates with previous research by Alhabash and Ma 2017, who observed a similar trend among university students, noting that Instagram and Snapchat were preferred over traditional platforms such as Facebook and Twitter [12]. Likewise, Kircaburun et al., 2018 [13] found Instagram to be among the most frequently used social media channels among university students in Turkey, second only to WhatsApp. The primary motivations for utilising Instagram, as identified by Sheldon and Bryant 2016 [14], encompassed surveillance, documentation, coolness, and creativity. This reflects the diverse approaches to digital communication within healthcare disciplines, as evidenced by the intriguing distinction in communication preferences among nursing, midwifery, and dietetics students in this study. Nursing and midwifery students may prioritise personal connections and informal interactions on social media platforms, leveraging platforms like Instagram to engage with peers and share personal experiences related to their field. In contrast, dietetics students’ preference for professional accounts suggests a focus on maintaining a professional image and disseminating evidence-based healthcare and nutrition information to a wider audience. These findings underscore the nuanced differences in digital communication practices among healthcare students, highlighting the importance of tailoring digital literacy interventions to meet the specific needs and preferences of students within different disciplines.

There has been a growing trend among dietitians and other healthcare professionals to establish professional accounts on social media platforms in recent years. This shift towards professional accounts reflects an appropriate means for healthcare professionals to showcase best practices in their respective fields. Social media can offer numerous benefits, including the dissemination of knowledge and skills, networking opportunities, raising awareness of the profession, and educating the public [15]. Both personal and professional social media accounts can expose trainees or registered healthcare professionals, and past online activities may have consequences, including professional scrutiny or investigation. Hence, it is significant to integrate education on digital professionalism into healthcare training programmes, emphasising upholding professional standards, privacy, confidentiality, and understanding the impact of digital footprints on reputations. By fostering a culture of digital responsibility, educators can prepare students for ethical and legal challenges in healthcare engagement.

Qualitative analysis of our data revealed social media as a prominent theme, with students emphasising the importance of ensuring that social media posts are evidence-based and include appropriate references. This sentiment aligns with recommendations from the Health and Care Professions Council [16], which reports the significance of considering the public’s perception of health professionals’ conduct and the importance of maintaining trust and confidence. 

Our study corroborates evidence from previous research [17] indicating that nutrition is one of the most prevalent topics for the dissemination of health-related misinformation on social media platforms. Furthermore, our findings highlight concerns raised by students regarding the proliferation of low-quality social media posts, particularly “before/after” images, which have been associated with potentially harmful outcomes such as increased body dissatisfaction. Interestingly, despite these concerns, a significant proportion of individual images shared by professional dietitians on Instagram focus on weight loss follow-up [18,19], suggesting the need for critical evaluation of content shared on social media platforms and its potential impact on public health perceptions and behaviours.

The study identified a range of training needs and aspirations among healthcare students, reflecting the evolving nature of digital healthcare practice. Nursing and midwifery students expressed a desire to enhance their proficiency in identifying and implementing digital tools for healthcare delivery, while dietetics students emphasised the importance of acquiring specialised skills such as Google Analytics and communication strategies for professional advancement. Furthermore, the study highlighted the role of dietitians in combatting misinformation and promoting evidence-based nutrition information online, underscoring the need for ongoing professional development in this area.

While our study primarily focuses on assessing digital competencies and social media usage among healthcare students, it is imperative to acknowledge the broader socio-economic factors that may impact students’ access to digital resources. Students facing constraints, including outdated hardware or limited access to the latest software, may encounter challenges in reaching optimal digital proficiency. This issue not only affects their ability to engage effectively with digital tools but also raises concerns about equity and inclusivity within healthcare education. Educational institutions must recognise and address these disparities by implementing initiatives to ensure equitable access to technology resources for all students. By providing support such as subsidised or loaned devices, access to software licenses, or technology training programmes, institutions can reduce the effects of digital poverty and empower students to develop the necessary digital skills for their future healthcare practice. Moreover, when implementing widening access programs in the healthcare sector, it is crucial for healthcare educators to consider factors including income levels and age-related digital reluctance. By acknowledging these factors, healthcare educators can tailor their programmes to address the diverse needs and backgrounds of aspiring healthcare professionals, fostering a more inclusive and diverse healthcare workforce. Future studies should comprehensively investigate insights into the challenges faced by students from economically disadvantaged backgrounds, as well as those experiencing age-related digital reluctance, and identify opportunities for improving support mechanisms to enhance their educational experiences and outcomes.

This study boasts several strengths that enhance the robustness and significance of our findings. One notable strength lies in the mixed-method approach employed, which facilitated a comprehensive exploration of digital competencies and social media usage among healthcare and dietetics students. By triangulating quantitative survey data with qualitative insights obtained through focus group discussions, our study achieved a multifaceted understanding of the subject matter, thereby enriching the depth and breadth of our findings. Moreover, the inclusion of participants from both nursing and midwifery disciplines, as well as dietetics students from various European countries, adds diversity and breadth to our sample, enhancing the generalisability of our findings to a broader population of healthcare students. Furthermore, the translation of the survey instrument into Turkish for participants in Turkey demonstrates our commitment to inclusivity and accessibility, ultimately enhancing the representativeness of our sample and minimising language barriers that may impede participation. 

While our study provides valuable insights into the digital competence and social media usage of healthcare and dietetics students, it is important to acknowledge several limitations. Conducting the survey exclusively in English may have restricted participation among individuals from European countries who do not speak English fluently. Although efforts were made to address this limitation by translating the survey into Turkish for participants in Turkey, the lack of translations for other European languages may have influenced the participation rates in those regions. Furthermore, the relatively limited sample size in the quantitative phase of our study may have implications for the generalizability of our findings. However, the inclusion of focus group discussions allowed for in-depth exploration of participants’ perspectives and experiences, thereby enriching the qualitative insights obtained. The recruitment of dietetics students for focus group discussions through social media platforms facilitated participant engagement, but it may have introduced a potential source of bias. Participants who were active users of social media platforms may have been more likely to become aware of the invitation and subsequently join the focus groups. This recruitment strategy could have inadvertently biassed the sample towards individuals with a higher level of digital literacy or greater familiarity with social media, potentially influencing the research findings. Another limitation to consider is the evolving landscape of technology since the inception of this study. Over the course of this study, significant advancements have occurred in the realm of digital technology, particularly concerning IT, chatbots, and AI. Regular surveys to assess the evolving literacy needs of healthcare professionals will be essential to inform training programmes and ensure that the workforce remains proficient in navigating the evolving digital landscape. Despite these limitations, our study contributes valuable insights into the digital competencies and social media behaviours of healthcare and dietetics students, paving the way for future research to further explore these important topics in diverse populations and settings.

## 5. Conclusions

The findings of this study demonstrate the critical importance of incorporating digital technologies and social media into healthcare and nutrition education and practice. It is evident that a robust curriculum in higher education, supplemented by ongoing lifelong learning courses and training, is essential to support healthcare professions in navigating the complexities of digital healthcare practice. However, further research is warranted to gain a deeper understanding of the specific needs within both higher education and practice settings. In addition to curriculum development, there is a pressing need to establish professional and ethical standards for digital nutrition communication. Prioritising the development of guidelines that ensure the credibility and accuracy of digital nutrition content for healthcare and nutrition students and professionals is vital. By addressing these needs and challenges, healthcare educators can better prepare students to thrive in an increasingly digitised healthcare landscape. By embracing emerging digital technologies, healthcare educators can empower students to navigate the complexities of modern healthcare practice, ultimately enhancing the delivery of quality patient care and improving health outcomes.

## Figures and Tables

**Table 1:** Main source of information for Nursing (n = 85), Midwifery (n = 58), and Dietetics Students (n = 54).

	Nursing (n = 85) n (%)	Midwifery (n = 58) n (%)	Dietetics (n = 54) n (%)
Academic classes/lectures	49 (57.6)	31 (53.4)	20 (37.0)
Research publications	27 (31.8)	18 (31.0)	15 (27.8)
Conferences	10 (11.8)	9 (15.5)	10 (18.5)
Seminars/webinars	16 (18.8)	12 (20.7)	13 (24.1)
Media (TV and podcasts)	33 (38.8)	26 (44.8)	5 (9.3)
Social media/blogs	43 (50.6)	27 (46.6)	6 (11.1)
Representatives from companies	0 (0)	0 (0)	0 (0)
Never informed before	9 (10.6)	8 (13.8)	0 (0)

**Table 2:** Digital competences that students desire to improve (Nursing Students, n = 85; Midwifery Students, n = 58; Dietetics Students, n = 54).

	Nursing (n = 85) n (%)	Midwifery (n = 58) n (%)	Dietetics (n = 54) n (%)
To use Microsoft Office	38 (44.7)	30 (51.7)	7 (12.9)
To learn about e-mail functions and properties	23 (27.1)	24 (41.4)	5 (9.2)
To learn video and image editing	46 (54.1)	32 (55.2)	8 (14.8)
To learn how to create infographics	15 (17.6)	9 (15.5)	3 (5.5)
To use professional digital networks (LinkedIn, ResearchGate, etc.)	29 (34.1)	22 (37.9)	5 (9.2)
The usage of social media to communicate health-related information	36 (42.4)	37 (63.8)	8 (14.8)
Able to identify and install software, programs, and applications for better healthcare/dietetic practice	49 (57.6)	40 (69.0)	10 (18.5)
Evidence-based implementation of digital tools in healthcare/dietetic practice	48 (56.5)	39 (67.2)	9 (16.6)

**Table 3:** Digital platforms, posting frequency, and account types to share healthcare/nutrition information (Nursing Students, n = 85; Midwifery Students, n = 58; Dietetics Students, n = 54).

	Posting Frequency n (%)			Account Type n (%)		
	High	Moderate	Low	Professional	Personal	Mix
**Facebook**						
Nursing	6 (7.1)	13 (15.3)	25 (29.4)	-	54 (63.5)	2 (2.4)
Midwifery	2 (3.4)	2 (3.4)	24 (28.2)	1 (1.7)	36 (62.1)	2 (3.4)
Dietetics	-	1 (1.9)	1 (1.9)	-	3 (5.6)	-
**Instagram**						
Nursing	10 (11.8)	27 (31.7)	22 (25.9)	2 (2.4)	67 (78.8)	3 (3.5)
Midwifery	3 (5.2)	10 (17.2)	34 (58.7)	1 (1.7)	45 (77.6)	3 (5.2)
Dietetics	2 (3.7)	4 (7.4)	1 (1.9)	4 (7.4)	3 (5.6)	1 (1.9)
**Linked In**						
Nursing	-	-	7 (8.2)	-	12 (14.1)	-
Midwifery	1 (1.7)	-	3 (5.2)	-	11 (19.0)	-
Dietetics	-	-	1 (1.9)	2 (3.7)	1 (1.9)	-
**YouTube**						
Nursing	4 (4.7)	2 (2.4)	9 (10.6)	1 (1.2)	26 (30.6)	1 (1.2)
Midwifery	1 (1.7)	-	12 (20.7)	-	17 (29.3)	2 (3.4)
Dietetics	-	-	-	-	-	-
**Research Gate**						
Nursing	-	-	5 (5.9)	-	6 (7.1)	1 (1.2)
Midwifery	3 (5.2)	-	3 (5.2)	-	7 (12.1)	1 (1.7)
Dietetics	-	-	-	-	-	-
**Twitter**						
Nursing	4 (4.7)	4 (4.7)	18 (21.2)	-	6 (7.1)	1 (1.2)
Midwifery	3 (5.2)	3 (5.2)	17 (29.3)	-	7 (12.1)	1 (1.7)
Dietetics	-	-	-	-	-	-

High posting frequency: seven or more posts per week. Moderate posting frequency: 1–4 posts per week. Low posting frequency: one post per month.

## Data Availability

The raw data supporting the conclusions of this article will be made available by the authors on request.

## References

[1] Qamar Z., Hagedorn-Hatfield R.L., Gray V.B., Shilts M.K., Cuite C.L. (2022). Re-envisioning Nutrition Instruction in Higher Education. J. Nutr. Educ. Behav..

[2] Dwivedi Y.K., Hughes D.L., Coombs C., Constantiou I., Duan Y., Edwards J.S., Gupta B., Lal B., Misra S., Prashant P. (2020). Impact of COVID-19 pandemic on information management research and practice: Transforming education, work and life. Int. J. Inf. Manag..

[3] Cerrato P., Halamka J. (2021). The Digital Reconstruction of Global Health. The Digital Reconstruction of Healthcare.

[4] Farid D. (2020). COVID-19 and Telenutrition: Remote Consultation in Clinical Nutrition Practice. Curr. Dev. Nutr..

[5] Mohamed Hashim M.A., Tlemsani I., Matthews R. (2022). Higher education strategy in digital transformation. Educ. Inf. Technol..

[6] Althubaiti A., Tirksstani J.M., Alsehaibany A.A., Aljedani R.S., Mutairii A.M., Alghamdi N.A. (2022). Digital transformation in medical education: Factors that influence readiness. Health Inform. J..

[7] Moorhead S.A., Hazlett D.E., Harrison L., Carroll J.K., Irwin A., Hoving C. (2013). A New Dimension of Health Care: Systematic Review of the Uses, Benefits, and Limitations of Social Media for Health Communication. J. Med. Internet Res..

[8] Helm J., Jones R.M. (2016). Practice Paper of the Academy of Nutrition and Dietetics: Social Media and the Dietetics Practitioner: Opportunities, Challenges, and Best Practices. J. Acad. Nutr. Diet..

[9] Torun E.D. (2020). Educational use of social media in higher education: Gender and social networking sites as the predictors of consuming, creating, and sharing content. Acta Educ. Gen..

[10] EFAD (2018). EFAD Academic Standards. https://www.efad.org/wp-content/uploads/2021/10/efad-academic-standards-revised-june-2018.pdf.

[11] Brown J., Morgan A., Mason J., Pope N., Bosco A.M. (2020). Student nurses’ digital literacy levels: Lessons for curricula. CIN: Comput. Inform. Nurs..

[12] Alhabash S., Ma M. (2017). A Tale of Four Platforms: Motivations and Uses of Facebook, Twitter, Instagram, and Snapchat among College Students?. Soc. Media Soc..

[13] Kircaburun K., Alhabash S., Tosuntaş Ş.B., Griffiths M.D. (2018). Uses and Gratifications of Problematic Social Media Use Among University Students: A Simultaneous Examination of the Big Five of Personality Traits, Social Media Platforms, and Social Media Use Motives. Int. J. Ment. Health Addict..

[14] Sheldon P., Bryant K. (2016). Instagram: Motives for its use and relationship to narcissism and contextual age. Comput. Hum. Behav..

[15] Vaos-Wootton K. (2024). Social media and professional practice. Netw. Health Dig..

[16] HCPC (2017). Guidance on Social Media. https://www.hcpc-uk.org/globalassets/resources/guidance/guidance-on-social-media.pdf.

[17] Wang Y., McKee M., Torbica A., Stuckler D. (2019). Systematic Literature Review on the Spread of Health-related Misinformation on Social Media. Soc. Sci. Med..

[18] Inan-Eroglu E., Buyuktuncer Z. (2018). What images and content do professional dietitians share via Instagram?. Nutr. Food Sci..

[19] Rounsefell K., Gibson S., McLean S., Blair M., Molenaar A., Brennan L., Truby H., McCaffrey T.A. (2020). Social media, body image and food choices in healthy young adults: A mixed methods systematic review. Nutr. Diet..

